# Congenital Paralysis of the Oculo-Motor of Both Sides

**Published:** 1885-10

**Authors:** R. Tilley

**Affiliations:** 125 State street


THE CHICAGO

MEDICAL JOURNAL AND EXAMINER.

VOL. LI. OCTOBER, 1885. NO. 4.

### ORIGINAL COMMUNICATIONS.

### CONGENITAL COMPLETE PARALYSIS OF THE OCULO-MOTOR OF BOTH SIDES, THE MOVEMENTS OF THE IRIDES AND THE ACCOMMODATION BEING INTACT. *By* DR. R. TILLEY.

[Read, and the patient exhibited, before the Chicago Medical Society, Sept. 7th, 1885.]

Complete paralysis of all the branches of the motor oculi of both sides, influencing the external movements of the eye in a boy of twelve years of age, is certainly not a common affection. Still less common, judging from the literature, is the above condition associated with the normal activity of the irides, and the function of accommodation intact. I refer to the present age of the boy, lest any should contend that I have not sufficient grounds for claiming for the case which I present to you a congenital origin. The mother informs me that a few days after his birth it was observed that the upper eyelids were never raised. In other words, that ptosis existed on both sides. She did not seem to be conscious, even about three months ago, of the peculiar condition of the muscles of the eye-balls. Whilst then the assertion of the mother, as to the existence of ptosis at this early age, is the most direct evidence I have to present, as to a part, at least, of the difficulty being of congenital origin, it is reasonable, however, to suppose that with a paralysis of the levators at this early age, the paralysis of the other branches of the same nerve existed at the same time. Moreover, if any marked change had occurred in the boy's eyes at any time after he began to notice much, the mother would have found it out. That the paralysis cannot be of recent origin, is evident from the failure of the common test. If I ask him to touch rapidly the tip of my finger, using either eye, he never fails to strike it exactly. I submit, then, for criticism the question of this being legitimately called a case of congenital origin, with such evidence as I have presented.

The question, of course, is here not one of paralysis or paresis of individual branches of one or both of the oculo-motors, but of complete paralysis of all the branches, excepting only the irides and the ciliary muscles. In looking up the literature, so far I have found little satisfaction; so that I am not in a position to form any estimate as to the frequency with which such a difficulty is observed. One tabulated form of the cases of paralysis of the muscles of the eye, as they were observed in Dr. Hermann Cohn's clinic, and published by Dr. Paul Schubert (p. 96), I find, out of one hundred and ninety-nine cases of paralysis, thirteen are said to have affected all the branches of the oculo-motor, leaving us to suppose that the iritic nerves, and those supplying the ciliary muscles, were also affected. We are, however, not informed whether both oculo-motors were affected or not. Out of these thirteen, in assigning the various causes, one only is said to be congenital and two unknown.

Alfred Von Graefe, in "Graefe und Sæmisch," when discussing the question of the paralysis of the oculo-motor, and speaking of the immunity of the irides and the ciliary muscle, says: "It is remarkable that sometimes when the oculo-motor is in other respects completely paralyzed, the characteristic mydriasis and paralysis of accommodation is not present." "Possibly," he adds, "in such cases there existed an anatomical peculiarity," stating, as the basis of his supposition, that Adamük* found, in three out of forty-two cases, the nerve fibres associated with the contraction of the pupil arising from the abducens instead of the oculo-motor.

With these introductory remarks, I present to you the patient, a boy of twelve years of age. His father and mother are both living. He had one brother, who died young. Separation of the parents will account for the fact that no other children followed this one. Under the circumstances, I cannot get any direct paternal history which would be of interest. Both of the children came quickly after marriage. There was nothing peculiar that I can elicit from the mother, in the way of sickness during the patient's childhood, only he was slow in walking. He is certainly not wanting in intelligence. He constitutes one member of a firm for the delivery of newspapers. In his school he has not done badly, especially when we take into consideration his disadvantage.

His appearance, attitude and gait present a perfect picture of the peculiarities of this affection. You will observe how both lids drop over the eyes, and that any effort to open or close them is effected by the frontalis and the corrugators. I will call attention, however, to a distinct crease which exists now in the external part of the lid, corresponding practically to the upper border of the tarsus. That crease, or fold, did not exist a month ago. I shall refer to that later. You will notice, also, that although he can stand with his head erect, in

*P. 56.

doing so he is not assuming the most advantageous position for the use of his eyes, on account of their being turned downwards by the action of the superior oblique muscles. To overcome this, he directs the head somewhat backwards. He can use either eye at will, although he prefers the left, a circumstance which is peculiar, as the visual acuity of the right is considerably greater. As I lift the eyelids, you will see that neither of them, with the head straight, serves to show him the way just in front of him; consequently, in walking, if he is using the right eye, he turns his head to the left, so as to bring the field of vision as nearly as possible in front of him; and if he is using his left eye, he turns his head to the right. At the same time, he combines with the sidewise direction a backward direction, to overcome the disadvantage of the eyes being turned downwards. All the movements of which his eyes are capable are confined to such as are possible from the antagonism of the external recti and the superior oblique muscles. Movements upwards, inwards and directly downwards are impossible. In inspecting the eyes you will see, also, that the left deviates more to the left than the right does to the right; consequently, when he walks, using his right eye, he turns his head less to the left than he turns his head to the right when he uses his left eye.

The distance to which the eyes deviate is so great that in no part of the visual field does he get double vision; even it such were possible, the difference in the visual acuity of the two eyes might prevent the recognition of double images. The deviation you will be able to estimate roughly by the position in which he holds his books when reading with the separate eyes. You will be able to observe for yourselves, as he comes around, that the irides are active, both for accommodation and from the mere influence of light.

His vision is far from normal. Distant vision for left, $\frac{6}{100}$; for right, $\frac{6}{36}$; and neither eye gets any advantage from glasses. For near vision he can read S. 0.8 at 6″ with either eye. The right does not serve him so well as the left. The left, however, is more divergent. The exact amount of accommodation which he posesses I have not estimated.

The ophthalmoscopic examination, about two months ago revealed nothing abnormal beyond a well defined small black spot on the inner and upper quadrant of the posterior surface of the left lens. I did not attempt to estimate the refraction by the ophthalmoscope.

The color sense is normal; the olfactory organ is perfect; taste and hearing excellent. I mention these particulars merely to show that their investigation has not been neglected.

As to the etiology of this particular case, I am strongly suspicious of the presence of specific trouble on the paternal side. The several points which are not conclusive, but of considerable importance, I prefer not to discuss at present. The circumstances are not propitious for investigation. I wish, however, to advance the historical testimony against my theory: Von Graefe, in Vol. vi, p. 72, "Graefe und Sæmisch," leads one to infer that he has not observed paralysis as a result of congenital specific trouble, or, at any rate, that it is very rare, and states that he does not find it referred to in a book on the special subject of inherited syphilis in its influence on the eye and ear, by Johnathan Hutchinson. As I may be able later to obtain more direct information bearing on the present case, I will leave the question without further discussion now.

As to the seat of the lesion, the matter is, of course, one of speculation. But the speculation is highly interesting. Its extent and uniformity alone, I think, excludes any conception of its being peripheral in origin. It would be folly to entertain the idea of any obstruction in the orbits. The meninges are the favorite seats of primary specific deposits, but were the meninges the chief source of trouble here, we should probably have had other associated difficulties, either of hearing, smell, or paralysis of other nerves. If, then, we exclude the peripheral origin, and exclude any obstruction in the orbit, if we do not find sufficient evidence of meningeal trouble we must seek for the lesion in the cerebral substance somewhere, either at the origin or course of the nerves before leaving the brain substance. Further speculation as to the location in the cerebral substance I leave to others. I will here call attention to one important circumstance which somewhat favors the view that the lesion is in the nerve substance. There is complete absence of the patellar reflex. Now, however guarded we may be in our views of the relation of the patellar reflex to nerve-tissue change, we must admit that they are frequently associated. We also know that paralysis of the ocular muscles are commonly associated with the development of loco-motor atéxia, of which the absence of the patellar reflex is one of the suspicious symptoms. He has none of the other symptoms, however, of loco-motor ataxia which I can elicit. Whatever theory we adopt as to the pathological condition, it is difficult to realize how the fibres supplying the iris and the ciliary muscle should have so uniformly escaped. It seems necessary to suppose the existence of some anatomical peculiarity, or the development of a vicarious action in the branch coming from the ophthalmic division of the fifth. It will be remembered that the short root from the motor-oculi unites with the long root of the ophthalmic division of the fifth to form the lenticular ganglion. I submit that it is not impossible that this otherwise sensory nerve may, under the circumstances, associated with a congenital defect, take upon itself functions such as would, under normal circumstances, be performed by the ordinary motor nerve. Or it may be, as Von Graefe suggests, that the abducens has in this case supplied the motor fibres.

My treatment you will in part have anticipated from the view I have taken of its etiology. I will, however, add that in addition to specific treatment I have been using electricity, not with any great expectation. The difficulty of applying it in the given case to the affected nerves is considerable. Nevertheless, I am applying it. Further, to leave no available effort untried, on finding there was marked phimosis, with considerable elongation of the prepuce, I circumcised him about three weeks ago, in order that if there was any influence associated with reflex action from that source I might remove it. A little circumstance, small as it is, I can not fail to mention, which has occurred since the circumcision. Six days after the operation, when the boy came down to my office, I was observing the crease I referred to above, in the upper eyelid, corresponding to the upper border of the tarsal cartilage, and searching mentally for a means of ascertaining, without putting a leading question, as to whether the mother had noticed this peculiarity, when she told me, as though anticipating my thought, that the crease or fold in the upper eyelid had developed since the operation.

The prognosis as to relief of the difficulty does not seem promising, but I must say that within the past week my expectation has greatly increased. The movements of the eyes are certainly greater.

125 State street.

